# Vascular Injuries: Trends in Management

**DOI:** 10.5812/traumamon.6238

**Published:** 2012-07-31

**Authors:** Mohd Lateef Wani, Ab Gani Ahangar, Farooq Ahmad Ganie, Shadab Nabi Wani, Nasir-ud-din Wani

**Affiliations:** 1 Department of Cardiovascular and Thoracic Surgery, Sher-i-Kashmir Institute of Medical Sciences, Srinagar, India

**Keywords:** Vascular System Injuries, Saphenous Vein, Anastomosis

## Abstract

Vascular injury presents a great challenge to the emergency resident because these injuries require urgent intervention to prevent loss of life or limb. Sometimes serious vascular injury presents with only subtle or occult signs or symptoms. The patient may present weeks or months after initial injury with symptoms of vascular insufficiency, embolization, pseudoaneurysm, arteriovenous fistula etc. Although the majority of vascular injuries are caused by penetrating trauma from gunshot wounds, stabbing or blast injury, the possibility of vascular injury needs to be considered in patients presenting with displaced long bone fractures, crush injury, prolonged immobilization in a fixed position by tight casts or bandages and various invasive procedures. iatrogenic vascular injuries constitute about 10% of cases in most series; however the incidence is an increasing trend because more endovascular procedures such as angioplasty and cardiac catheterization are being performed routinely.

Civilian trauma is more frequently seen in young males. However, it can occur at any age due to road accidents, firearms, bomb blasts and diagnostic procedures. Most of the time, civilian trauma causes less tissue damage. There is an epidemic of vascular injuries in Kashmir valley because of problems in law and order in the past two decades. This review deals with the topic in detail.

## 1. Introduction

Kashmir valley being a conflict zone having problems in law and order for ore than two decades has witnessed an epidemic of vascular injuries. Our hospital (Sher-i-Kashmir Institute of Medical Sciences) is the only tertiary care hospital where facilities for the management of vascular injuries are available catering a population of more than ten million people (1-4).

Most vascular traumas in military conflicts occur from fragments from ballistic weaponry such as artillery, rockets, grenades, and bombs. The development of high velocity weapons (such as artillery) cause massive tissue destruction that exceeds that from previous military weapons of equal size. Experimental studies of high velocity wounds show that a temporary cavity can be created nearly thirty times as large as permanent residual tract(5).This temporary cavitational effect injures the tissue beyond the actual path of the missile.

Vascular injuries can be divided into following groups:

• Spasm

• Thrombosis

• Contusion/Intimal flap

• Laceration/Transection

• A-V (arteriovenous) fistula

• Aneurysm and Pseudoaneurysm

• Arterial emboli

**Spasm:** Trauma to a vessel leads to the spasm of localized vasculature which causes decreased distal blood flow and can lead to functional damage distally.

**Thrombosis:** Any injury to the intima of an artery is followed by thrombosis over a period of time which cause partial or complete occlusion of a vessel or may undergo reversible segmental spasm.

**Contusion /Intimal Flap:** Concussive force or extra stretch causes a tear in the intima of the vessel. Small flap of <5mm may not significantly occlude blood flow but become a nidus for thrombosis. Large flap may protrude into the lumen of the vessel causing partial occlusion and symptoms of distal ischemia.

**Laceration/Transection:** Laceration and transection is produced most commonly by blunt trauma as well as by high velocity missiles which cause irregular tears in the vessel or segmental loss associated with other tissue injuries as well. A cleanly transected artery will often constrict and retract limiting blood loss. A longitudinal or badly lacerated vessel cannot limit blood loss and may produce greater blood loss. This type of injury comprises 80-85% of the cases (1).

**Arterio-Venous Fistula:** Injury to artery and adjacent vein may result in the development of a connection between the two with high pressure flow diverted towards low pressure (artery to vein), resulting in inadequate perfusion and distension of the veins. Any A-V fistula in central vessels cause congestive cardiac failure and presentation is usually late.

**Aneurysm and Pseudoaneurysm:** A true aneurysm contains all layers of vessel wall (intima, media and adventitia) and may be rarely produced by trauma. More commonly a pseudoaneurysm forms following trauma.

When hemorrhage from an injured vessel is stopped by surrounding fascia and other soft tissue, it is gradually encapsulated by fibrous tissue similar to adventitia in all properties. All this results in a thin walled capsule and goes on expanding due to higher arterial pressure forming a palpable pulsatile mass which can compress surrounding structures causing neuropathy, collapse of veins, erosion of bone or embolization of distal vessels, producing ischemia. The control of hemorrhage following vascular injury has been a prime concern to humans since the stone age ; various means such as compression, hot iron, cold and bandaging were used to stop the bleeding.

Following the initial contribution of Celsus, Galen and their contemporaries, the use of ligature was essentially forgotten for almost 1200 years. Throughout the Middle Ages cautery was used almost exclusively to control hemorrhage. Jerome of Brunswick an army surgeon, actually preceded Ambroise Pare in describing the use of ligature as the best way to stop hemorrhage. Ambroise Pare (6), with a wide experience in the surgery of trauma, especially in the battlefield, firmly established the use of ligature for control of hemorrhage from open blood vessels. In 1952, he did the first amputation above the line of demarcation which was earlier done below the line of demarcation. In seventeenth century Harvey’s monumental contribution describing the circulation of blood greatly aided the understanding of vascular injuries. In 1674 a military surgeon named Morel introduced a stick into the bandage and twisted it until arterial flow stopped (7).The screw tourniquet came into use shortly thereafter. In 1873 Freidrich von Esmarch, introduced his elastic tourniquet bandage for first aid use in the battlefield. His discovery permitted surgeons to operate electively on extremities in a dry, bloodless field.

The first successful ligation of the common carotid artery for hemorrhage control was performed in 1803 by Fleming; but it was not reported until 14 years later by Coley (1817) because Fleming died a short time after the operation was performed (8). A servant attempted suicide by slashing his throat. When Fleming saw the patient, who appeared exsanguinated, there was no pulse and the pupils were dilated. It was possible to ligate two superior thyroid arteries and one internal jugular vein. A laceration of the outer muscular layer the carotid artery was noted, as well as laceration of the trachea between the thyroid and cricoid cartilages. This allowed the drainage from the wound to enter the trachea, provoking violent seizures of coughing, although the patient seemed to be improving. Approximately one week following the injury, Fleming wrote”*On the evening of the 17th, during a violent paroxysm of coughing, the artery burst, and my poor patient was, in an instant, deluged with blood”. “In this dreadful situation I concluded there was but one step to take, with my prospect of success; mainly, to cut-down upon, and tie the carotid artery below the wound. I had never heard of such an operation being, performed; but conceived that its effects might be less formidable, in this case, than in a person not reduced by haemorrhage”*(8).

The wound rapidly healed following ligature of the carotid artery and the patient recovered.The importance of collateral circulation in preserving viability of limb after ligation has been well understood for centuries. The fact that time was necessary for the establishment of this circulation was well recognized. Halsted (1912) reported cure of an iliofemoral aneurysm by application of an aluminium band to the proximal artery without seriously affecting the circulation or function of the lower extremity (9).It was Alex Carel who performed the first successful end to end anastomosis in 1896. Ten years later, Goyanes used vein graft to bridge an arterial defect in 1906.

The successful repair of vascular injuries in the Korean conflict is a pleasant contrast to the experience of World War II, because of substantial progress in techniques of vascular surgeries accompanied by improvement in anesthesia, blood transfusion and use of antibiotics. Perhaps the most important was the rapid evacuation and resuscitation of wounded men. In addition, a thorough understanding of importance of debridement, delayed primary closure and antibiotics greatly decreased the hazards of infection (10, 11).

Historically, most patients who sustained serious arterial injury did not survive long enough to reach medical care provider; those who did generally had minor wounds. With advancement in the health care system, many seriously injured patients now arrive at the hospital (even those with very serious vascular injuries e.g. carotid vascular injury) and are salvaged. Vascular repair was first successfully performed in the early 1900. The usual surgical approach to a major vascular injury was simply ligation of the vessel and amputation of the limb (e.g. during the civil war popliteal artery injury resulted in a 100% amputation rate). In World War II, repair lowered the rate to 72%. During the Korean war, a policy of mandatory surgical exploration of all potential vascular injuries lowered the amputation rate to 32% for popliteal injuries. A policy of performing arteriography on all suspected vascular injuries combined with evolving vascular repair techniques during the Vietnam war reduced the amputation rate of these injuries to 15%. With current diagnostic and vascular repair techniques, almost all of these injuries can be revascularised, although a severely mangled limb may still need amputation.

A cardinal operative principle in managing major vascular injury is to obtain proximal and distal control of injured vessel before entering the aneurysm (5). In extremities as in neck, control is achieved using standard extensile vascular exposure techniques(12, 13). In the chest, control of vascular injury hinges on correct selection of a thoracotomy incision because incision provides access to different thoracic vascular compartments besides access to the heart. In the abdomen, the major vessels are located in the retroperitonium and therefore, exposure is based on operative maneuvers that mobilize the intraperitoneal viscera off the underlying retroperitoneal structures (14, 15).

Any patient with multiple systemic traumas may require stabilization of their injuries before vascular injuries are addressed e.g. haemoperitonium may take preference over these injuries, but sometimes vascular injury may be the most severe or the only injury which needs management. However missile cardiac injury always takes preference over other injuries as they are fatal if not timely addressed. Although vascular injury may be obvious based on presence of pulsatile arterial blood loss from the wound and presence of a cold, pale, pulse-less extremity yet certain patients may be asymptomatic. Despite all the measures, there might be a diagnostic dilemma and a challenging task for attending surgeon.

Historical knowledge states the mechanism and exact time of trauma because limitation of warm ischemia time warrants necessary repair of an arterial injury within six hours before irreversible ischemia occurs. The occupation and the handedness of the patient is relevant in that they will sometimes influence the aggressiveness of surgical approach. Comorbid medical ailment that may lead to complications of wound healing such as peripheral vascular diseases, diabetes mellitus and acquired immunodeficiency syndrome (AIDS), cancer or immunosuppressive drug intake are important to detect. Finally presence of symptoms of diminished supply of blood such as pain, numbness or weakness suggestive of nerve injury, and paresthesias are essential to elicit. Patients who are unable to provide a history or symptoms of ischemia reliably (e.g. intoxication, spinal cord injury or head injury) represent a diagnostic challenge.

Examination begins with palpation of pulses and detection of pulse deficit is although unreliable sign but very important suggestive sign and signal for further investigation rather than immediate surgery. A false positive pulse deficit may occur in shock, segmental vasospasm, compressive dressings and casts, congenital absence of pulse and pre-existing vascular diseases. False negative signs are found in case of strong collateral establishment. Based on the clinical examination, the patient is assigned to one of the three categories that dictate the diagnostic and management plan.

**Category 1 (Hard Signs):** These include pain, pallor, pulselessness, parasthesias, paralysis, pulsatile bleeding and large or expanding hematoma. If the patient shows these signs, he will have > 90% chance of vascular injury.

**Category 2 (Soft Signs):** These include a relatively diminished but palpable pulse, a nonexpanding hematoma and peripheral nerve injury; 30-35% of these patients will have vascular injury.

**Category 3 (Asymptomatic high risk):** These include penetrating wounds within 1 cm of major neurovascular bundle areas (e.g. axillary, femoral and carotid) and knee dislocations or severely displaced long bone fractures. Up to 15-20% of these patients have vascular injury.

## 2. Diagnosis

Vascular injuries are often supported by invasive and noninvasive tests when there is a diagnostic dilemma.

### 2.1. Non Invasive Test

**Hand Held Doppler:** This non-invasive tool amplifies sounds and has its own limitations. A change in the quality of pulse from triphasic to biphasic or monophasic suggests arterial occlusion.

**Ankle-brachial Index:** Ankle-brachial reflex <0.9-1.0 is an indication for exploration. It has also its own limitations and observer faults.

**B-mode Ultrasound:** This can visualize both arteries and veins directly. Small A-V fistula, intimal flap, pseudo-aneurysm are difficult to detect by this method.

**Duplex Ultrasound:** It is a combination of Doppler with B-mode ultrasound. It can detect vascular injuries with high specificity.

**Color Flow Doppler Ultrasound:** Here sounds are converted to digitalized visual signs. Flow towards the transducer is visualized as red a (arterial) and flow away from the transducer as blue (venous). Because the flow is in digital format, it can be quantified.

### 2.2. Invasive Tests

**Angiography:** This is technically difficult but has high specificity and was made a routine in the Vietnam war which reduced the amputation rate drastically. This is an expensive technique and requires mobilization of patients as well as an expert team. With development of non-invasive techniques, its use is now restricted to only a few situations.

**MRA (Magnetic Resonance Angiography):** A number of features of MRA make it well suited for the evaluation of vascular compromise. The principle advantage of MRA is that it can cover an extended vascular territory and provide information at the points in three dimensions (3D) space yet the modality is non-invasive. The 3D data sets obtained make it possible to display vessels of interest in a variety of formats and slices can be oriented in any projection format. MRA has been used primarily to obtain images of the vascular lumen, although interest is growing in the use of MRA to delineate disease process by imaging vessel wall.

MRA is of two types: 1. MRA plain study.2. Contrast enhanced MRA.

**Advantages of contrast MRA over plain MRA:** Total time required to collect data for 3D study is quite short. There is increased coverage, provided contrast material fills the vessels of interest, contrast-enhanced MRA can be used to cover a very large volume with excellent contrast-to-noise properties. Major benefit of contrast-enhanced MRA is that because of the signal strength, sequences can be applied with a high band width, yet an adequate signal-to-noise ratio. All MRA sequences benefit from reduced echo-times because they restrict the signal loss associated with disordered blood flow.

**DSA (Digital Subtraction Angiography):** This is taken as the standard of reference. This standard, however, may not be taken as “Gold Standard’’. Intra-arterial DSA can produce exquisite images with the newer system. It combines the advantage of requiring less contrast material with the capability of very fast frame rates, but the relatively small field of view has limited most DSA in the lower extremities to a supplemental role. Non-subtracted digitally acquired angiograms of both the entire lower extremities can be obtained with a single contrast injection when a stepping table is used. Incidence of arterial injuries in civilians in different situations is about 50% the upper extremity and 47% in the lower extremity.

## 3. Treatment

**Primary Anastomosis:** This is done when there is no or minimal segmental loss. Segmental loss puts the primary anastomosis at risk and there is a risk of thrombosis, or-blow out if the anastomosis is under tension.

**Reverse Saphenous Vein Graft:** If there is a segmental loss of >2cms.Saphenous vein should be harvested from the contralateral limb. The vein should be used upside down in arterial repair. There should be no tension on the suture line nor any kink in the graft.

**Lateral Repair:** If there is a lateral tear only. The vessel tear is sutured primarily at the tear site.
